# Access to primary care and computed tomography use in the emergency department

**DOI:** 10.1186/s12913-018-2958-4

**Published:** 2018-03-02

**Authors:** M. Fernanda Bellolio, Shawna D. Bellew, Lindsey R. Sangaralingham, Ronna L. Campbell, Daniel Cabrera, Molly M. Jeffery, Nilay D. Shah, Erik P. Hess

**Affiliations:** 1 Department of Emergency Medicine, Mayo Clinic, 200 First Street SW, Rochester, MN 55905 USA; 2 Department of Health Sciences Research, Division of Health Care Policy and Research, Mayo Clinic, Rochester, MN USA; 3 Kern Center for the Science of Heath Care Delivery, Mayo Clinic, Rochester, MN USA

**Keywords:** Diagnostic imaging, Electronic health records, Emergency service, Health services, Multidetector computed tomography

## Abstract

**Background:**

The decision to obtain a computed tomography CT scan in the emergency department (ED) is complex, including a consideration of the risk posed by the test itself weighed against the importance of obtaining the result. In patients with limited access to primary care follow up the consequences of not making a diagnosis may be greater than for patients with ready access to primary care, impacting diagnostic reasoning. We set out to determine if there is an association between CT utilization in the ED and patient access to primary care.

**Methods:**

We performed a cross-sectional study of all ED visits in which a CT scan was obtained between 2003 and 2012 at an academic, tertiary-care center. Data were abstracted from the electronic medical record and administrative databases and included type of CT obtained, demographics, comorbidities, and access to a local primary care provider (PCP). CT utilization rates were determined per 1000 patients.

**Results:**

A total of 595,895 ED visits, including 98,001 visits in which a CT was obtained (16.4%) were included. Patients with an assigned PCP accounted for 55% of all visits. Overall, CT use per 1000 ED visits increased from 142.0 in 2003 to 169.2 in 2012 (*p* < 0.001), while the number of annual ED visits remained stable. CT use per 1000 ED visits increased from 169.4 to 205.8 over the 10-year period for patients without a PCP and from 118.9 to 142.0 for patients with a PCP. Patients without a PCP were more likely to have a CT performed compared to those with a PCP (OR 1.57, 95%CI 1.54 to 1.58; *p* < 0.001). After adjusting for age, gender, year of visit and number of comorbidities, patients without a PCP were more likely to have a CT performed (OR 1.20, 95% CI 1.18 to 1.21, *p* < 0.001).

**Conclusions:**

The overall rate of CT utilization in the ED increased over the past 10 years. CT utilization was significantly higher among patients without a PCP. Increased availability of primary care, particularly for follow-up from the ED, could reduce CT utilization and therefore decrease costs, ED lengths of stay, and radiation exposure.

## Background

Computed tomography (CT) utilization has more than doubled over the past three decades, increasing in the number of studies performed as well as the total radiation dose per patient [1–3]. While this rapid growth was noted throughout various healthcare delivery settings from the 1990s to the early 2000s, studies have suggested that the overall growth of CT use has plateaued [4–6] While the growth of CT utilization appears to have slowed down or even stalled completely in alternate settings, this trend does not seem to apply to the emergency department [7, 8].

The widespread availability and use of CT as a diagnostic modality in the ED has likely led significant improvements in patient care [9]. Nevertheless, the rate of growth of utilization has called into question the value of these studies [10] the risks of overutilization, and the number of incidental findings [11]. The cost, psychological stress, and sequelae of further investigations of these incidental findings must be weighed against the benefit of the yield of early detection of true pathology.

CT carries a small but present risk to the patient of radiation induced malignancy, with the over 70 million studies performed every year estimated to result in approximately 29,000 future cancers [12, 13]. This effect is particularly worrisome in that it appears to preferentially affect children. Lastly, obtaining a CT scan and awaiting interpretation adds to patient length of stay, which may contribute to ED overcrowding as well as cost [14].

Drivers of the continued increased utilization of CT in the ED are many-fold and likely include systemic factors such as increased availability of scanners and improvements in technology as well as physician specific factors such as malpractice fear and different risk tolerance. Given the potential downsides of overutilization of CT in terms of resource utilization as well as radiation exposure, it is important to identify factors associated with variability in utilization in order to optimize practices.

The decision to obtain a CT scan is complex, involving multiple factors, including the likelihood that a patient has the diagnosis in question (pre-test probability), the inherent risk posed by a given test, the severity of the consequences of missing the diagnosis, and provider as well as patient risk tolerance [15]. CT utilization has been shown to vary according to provider specific factors, including risk tolerance level and geographical location [16, 17], as well as patient factors, such as language barrier [18].

We hypothesized that patient access to primary care also has an impact on the decision to obtain a CT Scan in the ED. For patients without access to primary care, it may be more detrimental to miss a diagnosis in its first presentation. For example, a provider may be more comfortable sending home a patient with abdominal pain without first performing a CT scan if they believe that the patient will be able to see another provider in 24–48 h, at which point there will be another opportunity for the correct, potentially lifesaving diagnosis to be arrived at. In contrast, for patients with limited access to care, the provider may reason that the patient is less likely to obtain follow-up evaluation, shifting the risk benefit ratio in favor of performing a CT scan during that visit and lowering the test-threshold.

Our primary objective was to determine if CT scans were less likely to be obtained in patients with access to primary care when compared to patients without such access. Specifically, we hypothesized that providers would be more likely to obtain a CT scan in patients without a primary care provider.

## Methods

We conducted a cross-sectional study of patients who presented to Saint Mary’s Hospital Campus Mayo Clinic, an academic, tertiary care emergency department. The Mayo Clinic ED provides care for approximately seventy-five thousand patients a year and serves a hospital with 1265 beds, receiving approximately four thousand transfers annually. Twenty percent of visits are by pediatric patients and the department features a dedicated pediatric section as well as serving a dedicated pediatric hospital with 85 beds including a pediatric intensive care unit and a neonatal intensive care unit. The radiology department performs and interprets more than one million exams yearly and features 20 CT scanners, including eleven 64-detector machines. Patients who presented to the emergency department from 2003 to 2012 and had a CT obtained in the ED were included. Every patient (or patient guardian) who presents to our center is asked for written permission to have their records reviewed for research purposes. A total of 7.5% of the patients did not provide this permission and were excluded. The institutional review board approved the research protocol.

Data were abstracted from the electronic medical record and an administrative database. We use an institutionally developed health record system across the enterprise. We have access to all visits within the main campus as well as the Mayo Clinic Health System. Our medical record automatically tracks and displays in the main screen, the primary care physician assigned to each patient within our institution, as well as outside institutions (if available). In our healthcare system, primary care can be available at our main institution (Mayo Clinic Rochester) as well as in the surrounding Health Care Systems sites.

CT scans obtained in the emergency department from 2003 to 2012 were identified in billing data using standardized Current Procedural Terminology, Fourth Edition (CPT-4) codes [19]. CT scans were organized by body region into four categories: “head,” “chest,” “abdomen,” and “other.” The category of “other” included a variety of uncommon scans examining the spine, extremities, neck and sinuses. The principal diagnosis for each CT scan was obtained using diagnosis codes from administrative billing data, and these diagnoses were tabulated. The ED disposition and admitting diagnosis were collected. We used the Agency for Healthcare Research and Quality’s Clinical Classification Software to organize the principal diagnoses into categories [20].

Demographic variables collected included date of birth, sex, chronic conditions, race, and presence or absence of a listed primary care provider. Patients were grouped by age into 6 categories: less than 18, 18 to 34, 35 to 49, 50 to 64, 65 to 79, and greater than 79 years of age. The primary and secondary diagnoses for each ED visit were obtained using diagnosis codes from administrative billing data. We used previously published algorithms- a modification of Hwang- to identify chronic conditions [21].

Our main outcomes were CT utilization rates per 1000 patients. Overall utilization trends were examined by year, body region, and age group over the study period. The availability from the ED for CT scans and other imaging modalities like ultrasound has remained stable during the study period. At our institution physicians and advance practice providers (nurse practitioners and physician assistants) can order CT imaging without consultation of a radiologist, and the access to imaging has been similar over time.

We report descriptive statistics with frequency counts and percentages. We assessed the association between clinical characteristic, particularly the presence or absence of a PCP, and whether a CT was obtained using logistic regression and report the associations as odds ratios with 95% confidence intervals (CI). Multivariable logistic regression models were constructed to evaluate the association between CT use during an ED visit and patient demographics. Variables were selected as covariates in the model according to their hypothesized influence on CT use. Linear regression was used to assess for trends by year in CT imaging (number of CTs per 1000 patients). All analyses were conducted using SAS statistical software, version 9.1 (SAS Institute, Cary, NC). Statistical significance was set at 0.05. We adhered to the STROBE guidelines for reporting observational studies [22].

## Results

A total of 595,895 ED visits, including 98,001 (16.4%) in which a CT was obtained, were included. Mean (SD) age of the study population was 41.6 (26.0) years, and 51.9% female. Fifty-five percent (328,609) of patients included had a PCP. Those with a PCP had a mean (SD) age of 36.2 (26.0) years and 45.8% were male, compared to 48.2 (24.3) years and 50.9% male for those without a PCP.

**Fig. 1:**
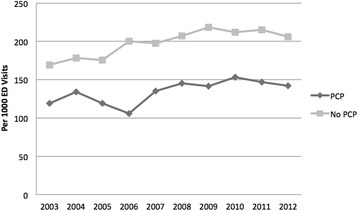
Computed Tomography use by year in patients with a primary care provider versus those without. Computer tomography (CT) per 1000 ED visits, 2003 through 2012, stratified by whether or not a patient had a known primary care provider (PCP). Throughout the study period patients without a PCP (No PCP) were more likely to have a CT scan

CT utilization per 1000 ED visits increased from 142.0 in 2003 to 169.2 in 2012 (19.2% increase, *p*–value for trend < 0.001). The number of ED visits during this time period remained relatively stable (55,241 visits in 2003 and 52,967 visits in 2012). CT utilization per 1000 ED visits increased from 169.4 to 205.8 over the study period for patients without a PCP (21.5% increase) and from 118.9 to 142.0 for patients with a PCP (19.4% increase; *p* < 0.001 for the rate of CT use in those with and without a PCP) Table 1. Patients without a PCP (OR 1.57, 95%CI 1.54 to 1.58; *p* < 0.001) were more likely to have a CT performed in the ED when compared to those with a PCP, as shown in Fig. 1.

**Table 1:** Trends in ED Computed Tomography Use by Study Year (2003–2012) and Patient Demographics

Year	2003	2004	2005	2006	2007	2008	2009	2010	2011	2012
Rate per 1000 ED visits with CT obtained	142.0	154.3	143.5	164.1	163.6	173.3	176.5	179.7	176.6	169.2*
Age										
< 18	56.9	55.1	52.3	56.1	48.2	49.0	44.0	40.2	36.6	33.5
18–34	136.5	146.0	130.8	149.9	142.6	149.3	155.9	155.7	144.8	143.6
35–49	155.7	174.0	156.7	179.2	177.2	189.1	198.2	191.4	198.4	183.5
50–64	176.9	192.1	176.3	198.6	210.6	221.0	222.6	219.9	226.6	218.6
65–79	198.2	204.3	205.1	229.1	230.3	243.6	257.4	271.1	262.9	262.0
> 79	226.5	235.7	226.3	249.7	269.6	283.7	279.4	288.1	285.5	304.0
Gender										
Female	145.1	160.0	142.9	166.7	167.5	175.0	175.1	182.3	180.9	174.2
Male	138.7	148.3	144.2	161.2	159.5	171.6	178.0	176.9	171.9	163.8
Primary Care Provider										
No	169.4	178.1	175.8	200.3	197.5	207.3	218.4	212.0	215.3	205.8
Yes	118.9	134.3	119.1	105.9	134.9	145.0	141.3	153.2	147.0	142.0

*p < 0.001 for the trend

An increase in CT use by year was seen in all patients older than 18 years of age and both genders (Table 1). After adjusting for age, gender, year of visit and number of comorbidities, patients without a listed PCP were still more likely to have a CT performed (OR 1.20, 95% CI 1.18 to 1.21, *p* < 0.001).

Computed tomography was performed in 4.8% of patients younger than 18 years of age and in 26.4% of patients older than 79 years (Table 2). The mean age (SD) for patients with a CT performed was 52.6 (23.0) versus 39.5 (26.0) for those without a CT (*p* < 0.0001). The odds of having a CT obtained increased by age [OR 3.4, 95% CI 3.3–3.5 for 18 to 34 years; OR 4.4 (95% CI 4.3–4.5) for 35 to 49 years, OR 5.2 (95% CI 5.1–5.4) for 50 to 64 years, OR 6.2, (95% CI 6.0–6.4) for 65 to 79 years, and OR 7.2 (95% CI 7.0–7.4) for patients older than 79 years. Female patients were as likely as males to have a CT ordered [16.7% versus 16.2%; OR 0.96 (95%CI 0.95–0.97)]. A greater proportion of Caucasian patients had a CT obtained compared to other ethnicities [17.1% White, 10.6% African American OR 0.57 (95% CI 0.55–0.60), 12.5% Hispanic OR 0.70 (95% CI 0.66–0.73), 13.9% Asian OR 0.78 (95% CI 0.74–0.83)].

**Table 2:** Emergency Department CT use by Study Year and Demographics

	No CT Ordered During ED Visit (*N* = 497,894)	CT Ordered During ED Visit (*N* = 98,001)	Overall (*N* = 595,895)	Odds of Receiving a CT (95% CI)
Age (years)				
Mean (SD)	39.5 (25.95)	52.6 (23.03)	41.6 (25.95)	
Age Group				
< 18	120,840 (95.2%)	6050 (4.8%)	126,890 (21.3%)	ref.
18–34	112,055 (85.4%)	19,105 (14.6%)	131,160 (22%)	3.41 (3.31, 3.51)
35–49	85,501 (82%)	18,802 (18%)	104,303 (17.5%)	4.39 (4.26, 4.53)
50–64	75,231 (79.2%)	19,741 (20.8%)	94,972 (15.9%)	5.24 (5.09, 5.40)
65–79	63,687 (76.4%)	19,714 (23.6%)	83,401 (14%)	6.18 (6.00, 6.38)
> 79	40,580 (73.6%)	14,589 (26.4%)	55,169 (9.3%)	7.18 (7.00, 7.41)
Patient gender				
F	257,526 (83.3%)	51,674 (16.7%)	309,200 (51.9%)	ref.
M	240,368 (83.8%)	46,327 (16.2%)	286,695 (48.1%)	0.96 (0.95, 0.97)
Year				
2003	47,397 (85.8%)	7844 (14.2%)	55,241 (9.3%)	ref.
2004	47,410 (84.6%)	8653 (15.4%)	56,063 (9.4%)	1.10 (1.07, 1.14)
2005	53,593 (85.6%)	8980 (14.4%)	62,573 (10.5%)	1.01 (0.98, 1.05)
2006	52,694 (83.6%)	10,342 (16.4%)	63,036 (10.6%)	1.19 (1.15, 1.22)
2007	52,006 (83.6%)	10,176 (16.4%)	62,182 (10.4%)	1.18 (1.15, 1.22)
2008	51,588 (82.7%)	10,818 (17.3%)	62,406 (10.5%)	1.27 (1.23, 1.31)
2009	50,534 (82.3%)	10,832 (17.7%)	61,366 (10.3%)	1.30 (1.26, 1.34)
2010	50,258 (82%)	11,010 (18.0%)	61,268 (10.3%)	1.32 (1.28, 1.37)
2011	48,411 (82.3%)	10,382 (17.7%)	58,793 (9.9%)	1.30 (1.26, 1.34)
2012	44,003 (83.1%)	8964 (16.9%)	52,967 (8.9%)	1.23 (1.19, 1.27)
Chronic Conditions				
Hypertension	39,825 (8.0%)	15,231 (15.5%)	55,056 (9.2%)	2.12 (2.07, 2.16)
Cardiac Dysrhythmias	22,000 (4.4%)	6572 (6.7%)	28,572 (4.8%)	1.56 (1.51, 1.60)
Asthma/COPD	22,101 (4.4%)	4550 (4.6%)	26,651 (4.5%)	1.05 (1.02, 1.08)
Coronary Atherosclerosis	20,565 (4.1%)	5614 (5.7%)	26,179 (4.4%)	1.41 (1.37, 1.45)
Depression	21,352 (4.3%)	3378 (3.4%)	24,730 (4.2%)	0.80 (0.77, 0.83)
Diabetes	18,718 (3.8%)	5756 (5.9%)	24,474 (4.1%)	1.60 (1.55, 1.65)
Hyperlipidemia	16,246 (3.3%)	5975 (6.1%)	22,221 (3.7%)	1.93 (1.87, 1.98)
Malignancies	15,034 (3.0%)	6569 (6.7%)	21,603 (3.6%)	2.31 (2.24, 2.38)
Congestive Heart Failure	12,154 (2.4%)	3000 (3.1%)	15,154 (2.5%)	1.26 (1.21, 1.31)
Personality Disorders	10,846 (2.2%)	1527 (1.6%)	12,373 (2.1%)	0.71 (0.67, 0.75)
No. Chronic Conditions				
None	343,771 (69.1%)	49,989 (51.0%)	393,760 (66.1%)	ref.
1–2	106,338 (21.4%)	31,191 (31.8%)	137,529 (23.1%)	2.02 (1.98, 2.05)
3+	47,785 (9.6%)	16,821 (17.2%)	64,606 (10.8%)	2.42 (2.37, 2.47)
ED Departure Status				
Admitted	85,750 (69.4%)	37,833 (30.6%)	123,583 (20.7%)	
Discharged	412,144 (87.3%)	60,168 (12.7%)	472,312 (79.3%)	
Race/Ethnicity				
White	425,872 (82.9%)	87,916 (17.1%)	513,788 (86.2%)	ref.
Black	26,599 (89.4%)	3146 (10.6%)	29,745 (5%)	0.57 (0.55, 0.60)
Hispanic	12,730 (87.5%)	1826 (12.5%)	14,556 (2.4%)	0.70 (0.66, 0.73)
Asian	8156 (86.1%)	1317 (13.9%)	9473 (1.6%)	0.78 (0.74, 0.83)
Other	24,537 (86.6%)	3796 (13.4%)	28,333 (4.8%)	0.75 (0.72, 0.78)
Primary Care Provider				
No	214,304 (80.2%)	52,982 (19.8%)	267,286 (44.9%)	1.57 (1.54, 1.58)
Yes	283,590 (86.3%)	45,019 (13.7%)	328,609 (55.1%)	ref.

All *p*-values are significant at the < 0.001 level, other than 2005 vs. Ref which was *p* = 0.46 and asthma *p* = 0.004

There was a 20.7% admission rate in this cohort. Admitted patients were more likely to have a CT obtained prior to admission, with 30.6% of those admitted having a CT performed versus 12.7% of those who were dismissed home (OR 3.0, 95% CI 3.0 to 3.1). After adjusting for age and gender, admitted patients were still more likely to have a CT performed (*p*-value for the model < 0.001). After adjusting by age and gender, patients without a usual source of care were more likely to be admitted to the hospital (*p*-value for the model < 0.001).

**Fig. 2:**
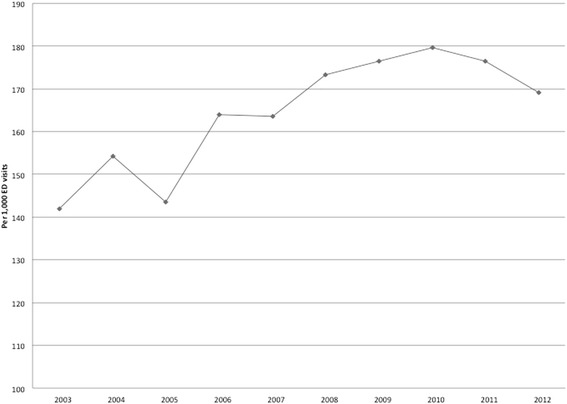
CT use per 1000 ED visits per year. CT imaging per 1000 ED visits, 2003 through 2012. The overall utilization rate increased from 142.0 CT scans per 1000 in 2003 to 169.2 per 1000 ED visits in 2013

**Fig. 3:**
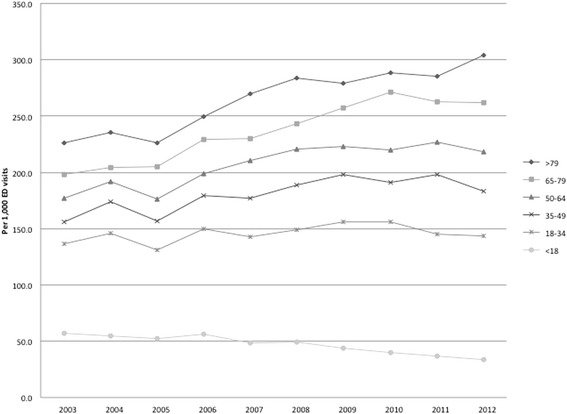
CT use per 1000 visits to the ED per year stratified by age. CT imaging per 1000 ED visits, 2003 through 2012, stratified by age. There was an increase in CT utilization in patients older than 18 and a reduction of CT utilization in children

Figure 2 shows trends in the number of visits in which a CT was obtained per 1000 ED visits during the study period. Figure 3 shows CT use per 1000 ED visits stratified by age. CT use increased throughout the study period in all ages with the exception of children (< 18 yrs. of age) in whom use decreased from 59.6 in 2003 to 33.5 in 2013 (43.8% reduction; *p*-value for trend < 0.001).

**Fig. 4:**
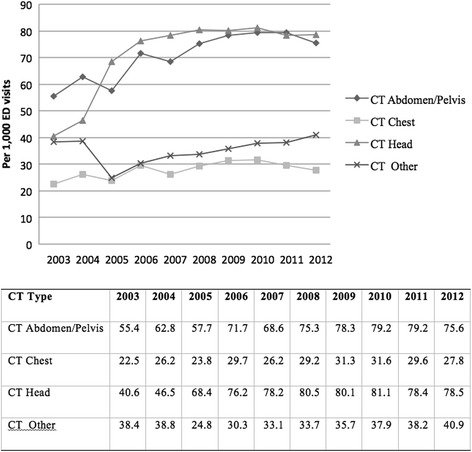
Trends in ED Computed Tomography Use by Year and CT type. CT imaging per 1000 ED visits, 2003 through 2012, stratified by CT type. CT Head had the highest utilization rate increase from 40.6 per 1000 ED visits in 2003 to 78.5 per 1000 ED visits in 2013

Figure 4 shows CT utilization rates throughout the study period by body region. CT Abdomen/Pelvis and CT Head are the most frequent types of CTs ordered. CT Head has the highest absolute increase from 40.6 per 1000 ED visits in 2003 to 78.5 in 2012, a 93.4% absolute increase. Headache was the most frequent indication for CT Head and was reported in 26%. Abdominal pain was the indication in 50% of CT scans of the Abdomen and Pelvis, followed by renal colic in 12%. Lower respiratory disease and chest pain accounted for 60% of the CT scans of the chest.

## Discussion

CT utilization per 1000 ED visits increased from 142.0 in the year 2003 to 169.2 in the year 2012, while the number of ED visits remained relatively stable in this time period. This is a 19% percent increase in the 10-year period and an absolute increase of 27.2 per 1000 ED visits. This confirms the results of the majority of previous studies, which have shown the continued growth in CT use in the ED without the plateau or even decrease in use that has been noted in non-ED settings [4–8]. However, our findings are in contrast to one single-center study that noted an initial spike and then dramatic downturn in CT utilization in the ED from 2002 to 2012 [23]. Notably, it appears that the emergency department described in that study underwent significant quality improvement efforts to optimize imaging utilization during that period, which may account for this outlier. The rate of increase noted in our study is less than that observed in similar reports, however our rate of utilization started out higher compared with other centers [2, 3]. We hypothesize that this is likely in part because of early adoption of radiology technology at our center, which introduced the first CT scanner in North America in 1973.

We did note a downturn in the CT utilization rate from 2010 to 2012, which coincides with a pattern described in similar studies [7, 24]. This downturn likely represents a change in coding in which CT of the abdomen and CT of the pelvis are now bundled together under one code. It is also possible that this downturn represents a trajectory towards a plateau or even decrease in CT utilization that continued after 2012, but we cannot determine this based on our dataset.

In contrast to their adult counterparts, CT utilization decreased in pediatric patients during the study period. Notably, our center includes a dedicated pediatric emergency department, which is true of less than 10% of hospitals in the United States [25]. Previous studies have found a decreasing rate of CT utilization in dedicated pediatric hospitals over a similar time period [26]. Our rate of CT utilization in the pediatric population was consistent with that previously reported. This trend is thought to be secondary to concerns about radiation, which are particularly heightened in the pediatric population who have longer remaining life-spans to realize the effects of radiation as well as greater radiosensitivity than their adult counterparts [27]. These concerns have spawned campaigns raising awareness of the potential risk of radiation, widespread use of highly sensitive clinical decision rules, and increased use of ultrasonography for a variety of indications, all of which have contributed to a decline of CT utilization in the pediatric population [28, 29]. This trend suggests that it may be possible to achieve decreased utilization in the adult population as well.

The demonstrated continued growth of CT utilization in adult patients during the time period of the study is part of a national trend which is multifactorial, likely spurred by increased availability of CT scanners, technological improvements and therefore broadened CT indications and capabilities, and changes in standard practice. While greater than 95% of EDs in the United States had access to a CT scanner by 2005, these machines varied in resolution and capability, features which may particularly affect the utilization of certain advanced studies, such as CT venography or angiography [3, 30]. As technology becomes universally available patient expectations as well as perceived standards of care adapt and utilization increases [31].

Providers are exposed to asymmetrical feedback, wherein positive CT findings that lead to a diagnosis, or conversely a missed diagnosis resulting from an un-ordered CT scan, have a disproportionate impact on clinical judgment when compared to the negligible impact of a negative study or uneventful outcome. Other factors that contribute to the decision to obtain a CT scan in the emergency department include requests by consulting services (for example: orthopedic surgery may request scans for operative planning), fear of malpractice, cost concerns, and the desire to enhance throughput.

It remains unclear and controversial as to whether emergency medicine physicians are overusing or underusing CT [32–34]. What is clear is that the rate of CT scans has undergone an explosive growth over the past two decades. A recent publication revealed that over 85% of surveyed emergency medicine physicians believe that we are doing too many diagnostic tests in the ED, listing fear of missing a low probability diagnosis and fear of litigation as top motivators for this trend [35]. This potential overuse of is problematic given the negative consequences of unnecessary CT, including increased costs, the threat of radiation induced malignancies, increased ED lengths of stay, false positive results, and incidental findings.

For these reasons, it is important to identify strategies to optimize and potentially decrease CT scan ordering. One way of accomplishing this it to identify and modulate factors associated with ordering a CT scan. Disposition decisions often rely on a clinician’s perception of the patient’s ability to comply with recommendations and obtain timely re-evaluation, and patients without such resources are more likely to be admitted to the hospital.

We found that patients without access to a primary care provider were more likely to have a CT scan performed in the ED (OR 1.57, 95%CI 1.54 to 1.58; *p* < 0.001). This association remained after adjusting for age, gender, and chronic conditions. Patients without a PCP were also more likely to be admitted to the hospital a finding also reported by Bindman and colleagues [36].

In addition to increased knowledge about best practices, malpractice reform, and integrated clinical decision support in electronic health records, increasing access to primary care and systematizing post emergency care follow-up are concrete interventions that can be made in a health system in order to optimize CT utilization and therefore reduce costs, radiation exposure, and ED length of stay.

This study has several potential limitations including its cross sectional design and use of administrative data, which vulnerability to miscoding and data entry errors common to all such studies. Further, this study was conducted at a single ED from a large academic center with a dedicated pediatric emergency department, thus the results may not be generalizable to other populations. Also caution should be taken when looking statistically significant results, and these should be in context of clinically significance.

However, there are several advantages to this study center over broader samples, especially in ascertainment of primary care status. This center features a robust electronic medical record, a highly integrated health system, and provides primary care for most of the patients in the surrounding area, all features which reduce likelihood of missing information based on the study design. Patients with primary care outside the health system were considered as not having a PCP, which is particularly important to note as we are a large referral center with many patients who travel long distances to seek care. This limitation is somewhat ameliorated by the consideration that access to a distant, and therefore less available, PCP is likely not as impactful in provider medical decision making as having a local PCP who could provide timely follow-up. Our emergency department receives about four thousand transfers every year, which has been relatively stable throughout the study period. Patients may have received CT studies at a transferring institution prior that would not be captured in our coding database, therefore potentially lowering our absolute rate of CT scan utilization.

Our study did not evaluate inter-physician variation or the appropriateness of the use of CT, although previous studies have shown dramatic variation in CT use among emergency physicians [37, 38]. Wong et al., using hierarchical logistic regression to facilitate comprehensive case-mix adjustment reported that only about 1% of the variability in ED imaging utilization was attributable to physicians. Predictors of imaging use were ED crowding, a prior ED visit, referral source to the ED, and ED arrival mode; physician-level factors including sex, years since graduation, annual workload, and residency training did not correlate with imaging use [39]. Lastly, our study population is less ethnically diverse and has a higher socioeconomic status than the overall U.S. population, with age, sex, and ethnic characteristics similar to those of the state of Minnesota and Upper Midwest [40].

## Conclusions

CT utilization in adult patients the Emergency Department has increased significantly over the past 10 years. Patients without a primary care provider were more likely to have a CT performed in the ED. Ensuring primary care access, particularly for ED follow-up, for the population might aid in optimizing CT utilization, therefore resulting in a decreases in costs as well as radiation exposure.

## Abbreviations

CI: Confidence intervals
CPT-4: Current Procedural Terminology Fourth Edition
CT: Computed tomography
ED: Emergency Department
OR: Odds ratio
PCP: Primary care provider
SD: Standard deviation
